# PTSD Symptoms Among Vietnam, Persian Gulf, and Post-9/11 Combat Veterans: Findings From VALOR

**DOI:** 10.1093/geroni/igaa057.2181

**Published:** 2020-12-16

**Authors:** Maria Kurth, Carolyn Aldwin, Richard Settersten

**Affiliations:** Oregon State University, Corvallis, Oregon, United States

## Abstract

Much is known about the mental health of combat Vietnam Veterans, but less is known about Persian Gulf and post-9/11 veterans and how they compare to those from earlier eras. Using data from an online survey of Oregon veterans, we examine how PTSD symptoms differ by combat exposure across these three cohorts. The sample (N=167, Mage=57.86, SD=12.09), was largely composed of White (88%), male (69%) Veterans. Most served in the Persian Gulf (41%), followed by Vietnam (36%) and post-9/11 (23%) eras. ANCOVAs showed significant cohort differences in PTSD, after controlling for severity of combat exposure and demographics (age, gender, education, income) (F(2, 157) = 4.24, p < .05). Post-9/11 veterans had significantly lower PTSD symptom severity than Vietnam-era veterans but were comparable to Persian Gulf. There were no cohort differences for noncombat veterans. Future research should investigate why Vietnam veterans continue to have worse mental health than younger veteran cohorts. Part of a symposium sponsored by the Aging Veterans: Effects of Military Service across the Life Course Interest Group.

